# Large-Scale Green Synthesis of Magnesium Whitlockite from Environmentally Benign Precursor

**DOI:** 10.3390/ma17040788

**Published:** 2024-02-06

**Authors:** Ruta Raiseliene, Greta Linkaite, Aleksej Zarkov, Aivaras Kareiva, Inga Grigoraviciute

**Affiliations:** Institute of Chemistry, Vilnius University, Naugarduko 24, 03225 Vilnius, Lithuania; ruta.raiseliene@chgf.stud.vu.lt (R.R.); greta.linkaite@chgf.vu.lt (G.L.); aleksej.zarkov@chf.vu.lt (A.Z.)

**Keywords:** whitlockite, large-scale synthesis, dissolution–precipitation synthesis

## Abstract

Magnesium whitlockite (Mg-WH) powders were synthesized with remarkable efficiency via the dissolution–precipitation method by employing an environmentally benign precursor, gypsum. Under optimized conditions, each 5.00 g of initial gypsum yielded an impressive amount of 3.00 g (89% yield) of Mg-WH in a single batch. Remarkably, no XRD peaks attributable to impurity phases were observed, indicating the single-phase nature of the sample. FT-IR analysis confirmed the presence of the PO_4_^3−^ and HPO_4_^2−^ groups in the obtained Mg-WH phase. The SEM-EDX results confirmed that Mg-WH crystals with homogeneous Ca, Mg, P, and O distributions were obtained. In previously published research papers, the synthesis of Mg-WH has been consistently described as a highly intricate process due to material formation within a narrow pH and temperature range. Our proposed synthesis method is particularly compelling as it eliminates the need for meticulous monitoring, presenting a notable improvement in the quest for a more convenient and efficient Mg-WH synthesis. The proposed procedure not only emphasizes the effectiveness of the process, but also highlights its potential to meet significant demands, providing a reliable solution for large-scale production needs in various promising applications.

## 1. Introduction

Bone defects can sometimes exceed their natural healing capacities and may require external intervention, which poses a substantial challenge to both the population and the health system [1,2]. In this context, synthetic implants with a chemical composition similar to that of native osseous tissue offer promising solutions [3,4,5]. Various synthetic calcium phosphate (CP) substitutes have been developed to promote the formation of new bone tissue within bone defects [6,7]. CPs have gained significant attention due to their exceptional biocompatibility, controlled biodegradability, osteoconductive properties, and potential for osseointegration [8,9]. Synthetic magnesium whitlockite (Ca_18_Mg_2_(HPO_4_)_2_(PO_4_)_12_, Mg-WH) is a promising bone substitute material that promotes bone formation under physiological conditions [10,11]. Although the abundance and distribution of Mg-WH within bone tissue remains a subject of ongoing research and debate, magnesium is recognized as an essential element in the human body that influences various physiological functions [12,13,14,15]. To our knowledge, the intricate synthesis of Mg-WH has been consistently described as highly complex [16,17]. This complexity is primarily attributed to the distinctive formation characteristics of the material, which necessitate precision within a narrow pH and temperature range. Moreover, Mg-WH, which is a thermally unstable phase, is difficult to synthesize using conventional high-temperature methods [8,18]. Upon annealing above ca. 600 °C, this material decomposes by releasing water and transforming into a mixture of Mg-substituted β-Ca_3_(PO_4_)_2_ and Ca_2_P_2_O_7_. A recent study by Kizalaite et al. [18] describes the thermally induced degradation of Mg-WH in detail. In this regard, the dissolution–precipitation synthesis method stands out as a viable technique for the fabrication of CP materials at lower temperatures, offering a promising solution to overcome the thermal challenges associated with Mg-WH syntheses [19,20].

The need for a large-scale synthesis of Mg-WH has been emphasized in previous studies [21,22]. Therefore, the objective of our study was to develop a simple and cost-effective synthesis route for the preparation of substantial amounts of Mg-WH powders. It should be noted that the ability to obtain a sample of high purity is crucial to ensure the reliability and reproducibility of subsequent investigations and applications, positioning the designated synthesis method as a robust approach for acquiring CP materials. Our approach focusses on the use of environmentally benign gypsum (CaSO_4_·2H_2_O) powder as a precursor, aligning with contemporary efforts to promote sustainability and reduce the environmental impact of material synthesis processes. Through this study, we aimed to contribute to the sustainable development of bone substitute materials that can facilitate more effective and accessible bone healing and restoration procedures, potentially improving the quality of life of people facing bone-related health challenges.

## 2. Materials and Methods

**Figure 1:**
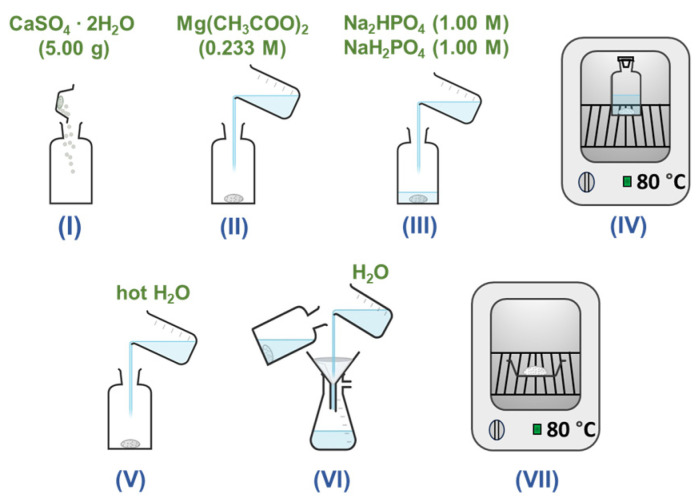
Schematic representation of the large-scale synthesis of Mg-WH powder. Gypsum powder was mixed with Mg(CH_3_COO)_2_, Na_2_HPO_4_, and NaH_2_PO_4_ solutions of stages I–III in a reaction bottle and placed in an oven (stage IV). After synthesis, the product was decanted, washed with hot water (stage V), vacuum-filtered (stage VI), and dried in an oven (stage VII).

Gypsum (calcium sulfate dihydrate, 99%, Sigma-Aldrich, St. Louis, MO, USA), magnesium acetate tetrahydrate (Mg(CH_3_COO)_2_·4H_2_O, 98%, Roth, Karlsruhe, Germany), disodium hydrogen phosphate (Na_2_HPO_4_, 98%, Merck, Darmstadt, Germany), and sodium dihydrogen phosphate (NaH_2_PO_4_, 99%, Merck) were used as starting materials for the fabrication of Mg-WH powders via a dissolution–precipitation reaction. The sequential depiction of the key synthesis stages is shown in Figure 1.

To outline the synthesis process, an initial 5.00 g portion of gypsum was carefully placed in a glass bottle. Subsequently, 31.25 mL of a 0.233 M magnesium acetate solution was added to the bottle. The reaction vessel was then filled with a mixture containing 250.0 mL of each 1.00 M Na_2_HPO_4_ and 1.00 M NaH_2_PO_4_. The pH of the resulting mixture was determined as 6.2. Such a pH value promotes the formation of Mg-WH and prevents the formation of other CPs, because pH is one of the key factors influencing the precipitation of particular CP phases. The sealed bottle was subsequently placed in an oven at 80 °C for 24 h, 48 h, and 72 h, allowing the reaction to progress. After synthesis, the liquid phase was decanted from the bottle, and the resulting powder was rinsed with 500 mL of hot (~80 °C) deionized water, followed by several additional rinses with 250 mL of room-temperature deionized water. Finally, the vacuum-filtered product was dried at 80 °C for 2 h. This meticulous procedure ensures precise control and sequential execution of each stage, contributing to the reproducibility and reliability of Mg-WH powder synthesis.

The prepared samples were characterized through powder X-ray diffraction (XRD) using a Rigaku MiniFlex II diffractometer with Cu Kα radiation (λ = 1.541838 Å). The diffraction data were obtained by scanning in the 2θ range of 10–60° at a scan speed of 2°/min. The phases obtained in this study were subjected to a thorough semi-quantitative analysis, a process intricately executed through the normalized corundum reference intensity ratio (RIR) method facilitated by the Match! (version 3.13; Dr. Holger Putz, Crystal Impact, Bonn, Germany). The analysis of the XRD pattern (measured in the 2θ range of 10–70°) was carried out using the Le Bail method with the help of FullProf software (https://www.ill.eu/sites/fullprof/php/programs.html) [23]. Standard lanthanum hexaborate (LaB_6_) was measured to acquire instrumental broadening of the diffractometer, which is crucial to eliminating its contribution from the width of the experimental diffraction peak and ensuring precise crystallite size [24]. To assess the crystallinity of the synthesized Mg-WH sample, accounting for the mixture of crystalline and amorphous phases in the experimental XRD pattern, the sample was analyzed using a zero-background Si sample holder with the help of the aforementioned XRD apparatus and Match! software (https://www.crystalimpact.com/match/). Fourier transform infrared spectroscopy (FT-IR) was performed using an Alpha spectrometer (Bruker, Inc., Ettlingen, Germany) in a wavenumber range from 1400 to 450 cm^−1^, with a resolution of 4 cm^−1^. The thermal decomposition of the sample was examined through thermogravimetric analysis and differential scanning calorimetry (TG-DSC) using a Perkin Elmer STA 6000 Simultaneous Thermal Analyzer (Pittsburgh, PA, USA). Approximately 10 mg of dried sample was heated from 25 °C to 900 °C at a controlled heating rate of 10 °C/min, all within a dry flowing air environment (20 mL/min). The product morphology was analyzed using field-emission scanning electron microscopy (SEM, SU-70, Hitachi, Tokyo, Japan). Energy-dispersive X-ray (EDX) analysis of the sample was performed using an SEM Hitachi TM 3000. The specific surface area was measured through the Brunauer–Emmet–Teller (BET) method under vacuum for degassing at 120 °C through N_2_ adsorption–desorption isotherm (at 77 K) using a Tristar II instrument (Norcross, GA, USA). The pore size distribution of the material produced was obtained using the Barrett–Joyner–Halenda (BJH) method.

## 3. Results and Discussion

**Figure 2:**
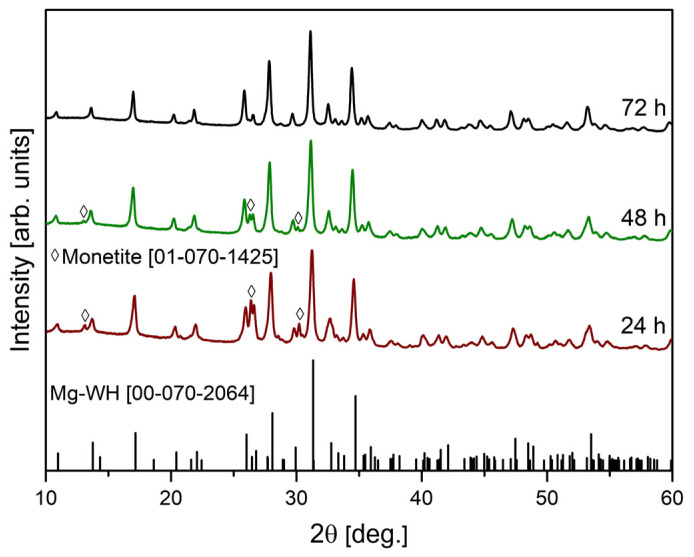
XRD patterns were measured for samples synthesized for various reaction durations, specifically 24 h, 48 h, and 72 h. The vertical lines below depict reflections from the standard XRD pattern of Mg-WH, according to #00-070-2064.

To investigate the time required for the formation of the Mg-WH phase, the reaction time was gradually extended from 24 h to 72 h, maintaining a constant synthesis temperature of 80 °C. The composition of the obtained samples was confirmed by analyzing the powder XRD patterns shown in Figure 2. When examining the XRD patterns of the samples synthesized for durations of 24 h and 48 h, a mixture of anhydrous calcium hydrogen phosphate (DCPA, CaHPO_4_; #01-070-1425) and the Mg-WH phase (#00-070-2064) were obtained. DCPA, due to its reactivity and solubility, is a valuable precursor material, allowing for the synthesis of various phases of CP, including calcium hydrogen phosphate dehydrate (CaHPO_4_·2H_2_O), calcium phosphate cements, and hydroxyapatite [25,26]. DCPA is known to be the most stable CP phase in solutions with a pH less than 5 [27]. However, it should be noted that DCPA stability is modulated in the presence of Mg^2+^ ions [28]. The appearance of the Mg-WH formation under magnesium-rich and mildly acidic and magnesium-rich pH conditions confirms the critical influence exerted by Mg^2+^ ions on the stability of DCPA [28]. This influence establishes the requisite conditions for the successful realization of the Mg-WH phase from gypsum via the DCPA phase within the synthesis parameters implemented in our study. In particular, as shown in Figure 2, the amount of DCPA diminished over time from 24 to 48 h with a simultaneous increase in the Mg-WH content. To investigate the crystalline phases present in these samples and to precisely quantify their compositions, the normalized corundum reference intensity ratio (RIR) method was employed. The results obtained are summarized in Table 1.

It was revealed that the 24 h sample exhibited a composition of 35 wt.% DCPA and 65 wt.% Mg-WH, while the 48 h sample demonstrated a distinctive composition of 22 wt.% DCPA and 78 wt.% Mg-WH. Extending the reaction time to 72 h revealed the disappearance of the DCPA phase in the XRD patterns, while the peaks associated with Mg-WH prevailed. Importantly, the discernible absence of any peaks associated with other crystalline phases, including precursor phases or intermediate compounds, in the XRD pattern confirmed the high purity of the prepared sample. Therefore, using the described synthesis procedure, 3.00 g of a single-phase Mg-WH structure sample was obtained in a single batch. This observation underscores the efficacy of the described synthesis procedure, demonstrating its capability to yield a large quantity of single-phase Mg-WH.

**Figure 3:**
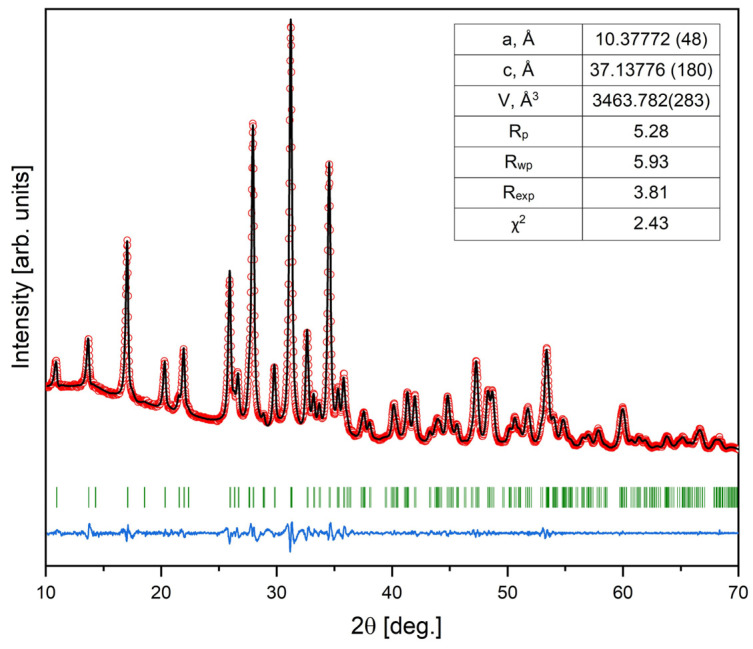
XRD pattern with the fitting curve through Le Bail refinement (red circles represent experimental points, and the solid line represents refined data; the blue line shows the difference between experimental and refined data; the 2θ positions marked in green are the allowed Bragg peaks) of 72 h synthesized Mg-WH.

XRD analysis of the 72 h synthesized sample through Le Bail fitting matched well the standard provided by the International Centre for Diffraction Data No.: 00-070-2064 and, in turn, aligned with the rhombohedral Mg-WH crystal structure with the R3c space group (#161) (see Figure 3) [29]. Here, it should be noted that Mg-WH might be conceptualized as a material in which magnesium potentially substitutes calcium in the structure of synthetic β-Ca_3_(PO_4_)_2_. Despite the apparent similarity in the XRD patterns of these materials, a profound examination through advanced analytical techniques of their crystal structures revealed substantial disparity [29,30].

The XRD data were further analyzed for the determination of the lattice parameters. The refined lattice parameters, *a* = 10.37772(48) Å and *c* = 37.13776(180) Å, are in good correlation with the values provided in the literature [18]. Furthermore, as shown in Figure 3, the observed XRD pattern of the Mg-WH sample aligns closely with the calculated one.

It is important to note that the XRD pattern observed for the Mg-WH sample exhibited diffraction peaks of notable widths. Furthermore, it was determined that the synthesized Mg-WH powder was composed of crystallites with an average size of 34 nm. A comprehensive analysis was conducted to assess the crystallinity, considering the coexistence of the crystalline and amorphous phases in the XRD pattern. Evaluation revealed a degree of crystallinity of 78%. It is well known that the crystallinity of CPs significantly influences the dissolution behavior of a material [31]. Typically, more crystalline regions exhibit slower dissolution rates than their amorphous counterparts, affecting the performance of the material in biological or environmental settings. This understanding is crucial for predicting the long-term stability and potential applications of synthesized materials.

**Figure 4:**
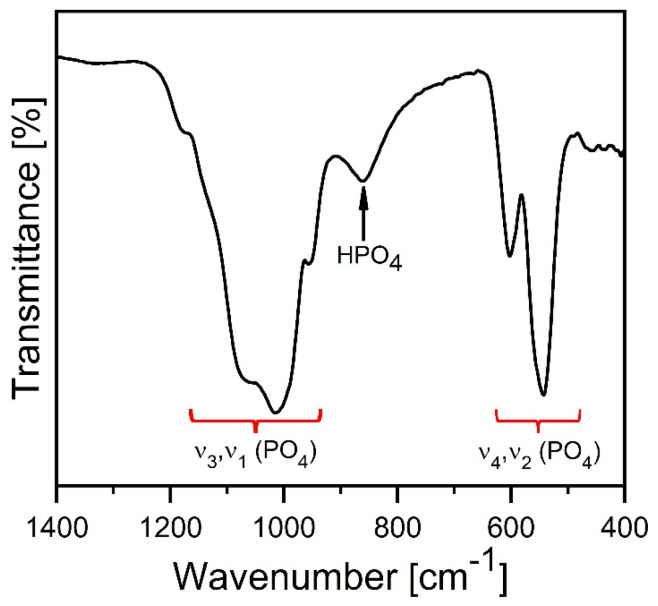
FT-IR spectrum of the 72 h synthesized Mg-WH.

FT-IR analysis was performed to confirm the structure of our 72 h synthesized Mg-WH sample (Figure 4). The observed spectral characteristics are indicative of distinct vibrational modes associated with the PO_4_^3−^ and HPO_4_^2−^ groups. A set of vibrations at 1171 cm^−1^, 1134 cm^−1^, 1064 cm^−1^, 1013 cm^−1^, and 954 cm^−1^ were associated with the stretching vibrations of the P–O bonds, denoted as ν_3_ and ν_1_, respectively [32]. A wide absorption band centered at 861 cm^−1^ was observed, indicating the stretching of the P–O(H) bond intrinsic to the crystal structure of Mg-WH [32]. The bands observed at 601 cm^−1^, 542 cm^−1^, and 459 cm^−1^ were associated with the bending vibrations ν_4_ of the P–O and ν_2_ (O–P–O) vibrations, respectively [33].

**Figure 5:**
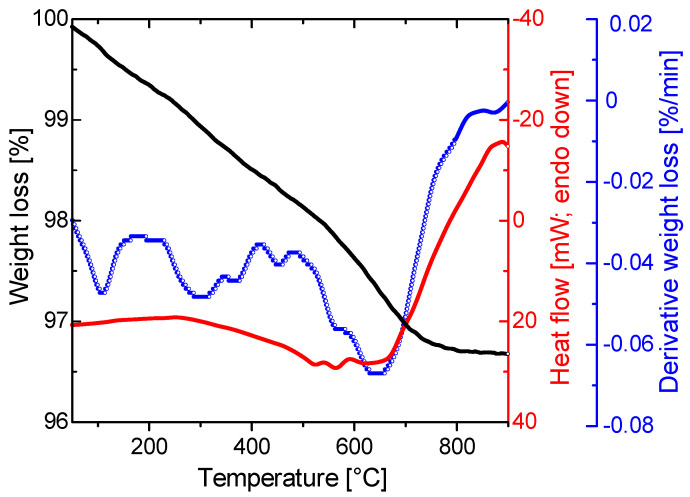
TG/DSC curves of the 72 h synthesized Mg-WH.

The thermal decomposition behavior of the 72 h sample was investigated through simultaneous TG-DSC measurements. The TG-DTG-DCS curves of the analyzed sample are shown in Figure 5. In particular, Mg-WH was predicted to undergo dehydration and condensation of the HPO_4_^2−^ group at around 600 °C, resulting in the formation of magnesium-substituted β-TCP ((Ca,Mg)_3_(PO_4_)_2_), Ca_2_P_2_O_7_ (calcium pyrophosphate), and water, according to the predicted chemical pathway elucidated in a previous study [20]. This process is associated with a theoretical weight loss of less than 1 wt%. Notably, the synthesized Mg-WH was quite stable up to 500 °C. Upon gradual heating of the 72 h synthesized sample, a continuous weight loss was observed, reaching completion at approximately 750 °C, with a total mass loss of approximately 3 wt%. The observed disparity in weight loss implies the existence of absorbed water in the as-prepared material, which is likely the result of the physically captured water on the sample surface during the synthesis process.

**Figure 6:**
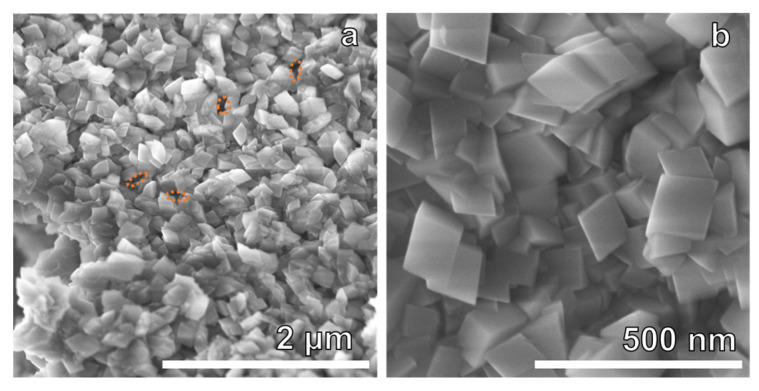
(**a**,**b**) SEM images of the 72 h synthesized Mg-WH.

The microstructure and surface morphology of the 72 h synthesized Mg-WH were investigated through SEM. Figure 6a,b show the SEM images of the sample. It can be seen that the surface of the sample is composed of uniform, rhombohedrally shaped crystals, featuring pointed tops, sharp edges, and dimensions within the range of approximately 75 to 150 nm. In particular, the observed particle growth mechanism appears to align with Ostwald ripening rather than with continuous nucleation. The ripening of Ostwald facilitates the dissolution of smaller particles upon synthesis, leading to the growth of larger rhombohedral crystals [34,35]. A small number of pores also formed during synthesis, as indicated by the highlighted orange ovals in Figure 6a. On closer examination at higher magnifications, as shown in Figure 6b, the flat surfaces of each particle were apparent, and no discernible impurities were observed. The well-defined rhombohedral shape is characteristic of WH crystals and agrees well with previously reported studies [16,36].

**Figure 7:**
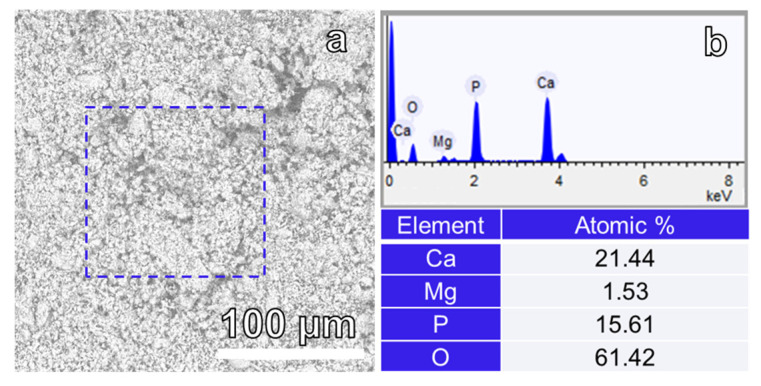
(**a**) Hitachi SEM image acquired with an SEM 3000 and (**b**) output of EDX spectra and atomic concentration of Ca, Mg, P, and O of the 72 h synthesized Mg-WH.

The elemental composition of the 72 h synthesized sample was examined using EDX spectroscopy to validate the homogeneity of the elemental distribution within the fabricated sample [37]. The EDX of the elemental analysis results showed the presence of calcium (Ca), magnesium (Mg), phosphorus (P), and oxygen (O) in the Mg-WH sample (see Figure 7). Quantitative analysis yielded atomic concentrations of Ca:Mg:P:O as 21.44:1.53:15.61:61.42 in the Mg-WH sample, closely aligned with the atomic concentration values for stoichiometric Mg-WH (Ca_18_Mg_2_(HPO_4_)_2_(PO_4_)_12_), which are 20.00:2.22:15.55:62.22. As suggested in [38], the inherent flexibility of the Mg-WH structure allows for variation in the calcium-to-magnesium ratio. Consequently, our synthesized sample exhibited a slightly reduced magnesium content compared to that of the stoichiometric Mg-WH, reflecting the adaptability of the Mg-WH structure to accommodate subtle compositional variations. Notably, the absence of sulfur (S) in the EDX spectrum served as a distinctive indicator, confirming the absence of precursor gypsum in the final sample. This absence underscores not only the purity of the Mg-WH phase, but also the effectiveness of the chosen synthesis route.

**Figure 8:**
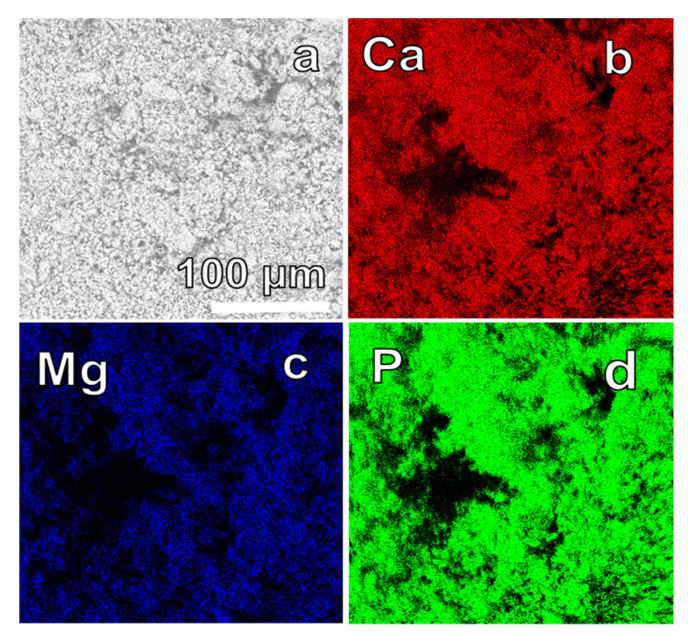
(**a**) SEM image acquired with a SEM Hitachi TM 3000 and corresponding elemental distribution EDX mapping of Ca (**b**), Mg (**c**), and P (**d**) of the 72 h synthesized Mg-WH.

Elemental mappings of Ca, Mg, and P are illustrated in Figure 8a–d. The results obtained depicted a consistent and uniform distribution of elements throughout the entire sample. In particular, there were no observable indications of segregation or the formation of additional phases, signifying a high level of homogeneity in the elemental composition.

**Figure 9:**
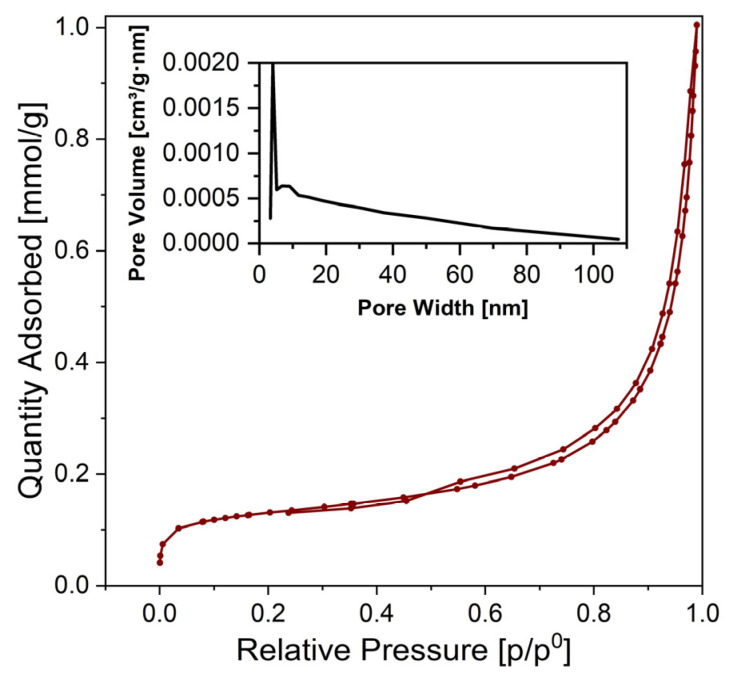
N_2_ adsorption–desorption isotherms of 72 h synthesized Mg-WH sample. The inset shows the pore size distributions obtained through the BJH method.

Figure 9 shows the N_2_ adsorption–desorption isotherm of the 72 h synthesized Mg-WH sample.

In the very low relative pressure range, the adsorption curve demonstrates concavity relative to the p/p^0^ axis, transitioning into a semi-linear segment in the middle section and increasing rapidly at p/p^0^ > 0.6. At a high relative pressure region of the isotherm, the adsorption curve exhibits convexity toward the p/p_0_ axis, with the amount of adsorbed N_2_ capable of increasing without limits when p/p^0^ equals 1. In particular, adsorption did not reach saturation (even when reduced to a mere inflection point), which is due to the presence of macropores in the sample [39]. In the course of the analysis, the disparity between the N_2_ adsorption and desorption curves becomes visible. This is particularly evident as a narrow gap between the adsorption and desorption branches, accompanied by a discernible hysteresis loop upon reduction of pressure [39]. The curves illustrated in Figure 9 show mixed types of II and IV isotherms characterized by a distinctive H3 hysteresis loop [39,40]. Typically, non-porous and macroporous adsorbents (i.e., materials in which the pores are greater than 50 nm) show type II isotherms, while type IV irreversible isotherms are specified by mesoporous adsorbents (i.e., materials with pores of widths between 2 nm and 50 nm) [39]. Loops of the H3 type are formed by non-rigid aggregates of platy particles that give rise to slit-shaped pores that arise from the stacking of crystal particles [39]. The pore size distribution curve (see inset in Figure 9) reveals a relatively narrow distribution of pore sizes, with a predominant peak reaching ~4 nm, corresponding to the mesopores [40]. The specific surface area (S_BET_) calculated using the BET equation was 10 m^2^ g^−1^ for the 72 h synthesized Mg-WH sample.

## 4. Conclusions

In this work, we present a simple and cost-effective dissolution–precipitation method for the green and large-scale production (3.00 g per synthesis) of Mg-WH powder. The proposed synthetic procedure utilizes gypsum as an environmentally friendly synthesis precursor, contributing to both simplicity and environmental sustainability in material processing. Our study demonstrates the controlled synthesis of the Mg-WH phase through systematic variation of the reaction time. XRD analysis reveals a transition in composition, confirming the successful formation of Mg-WH from gypsum via the DCPA phase. The absence of peaks related to other crystalline phases in the XRD pattern confirms the high purity of the prepared sample. The determined lattice parameters, *a* = 10.37772(48) Å and *c* = 37.13776(180) Å, are in good agreement with the literature data. FT-IR analysis further validates the crystal structure, while the observed well-defined rhombohedrally shaped crystals with dimensions of 75–150 nm and clean surfaces underscore the quality of the synthesized Mg-WH. Importantly, the evaluation of crystallinity reveals an average crystallite size of 34 nm and a degree of crystallinity of 78%. A small amount of pores and voids has also formed on the surface of Mg-WH during synthesis. This was also confirmed through BET measurements. Additionally, elemental analysis through EDX spectrometry confirms the homogeneous distribution of elements within the sample, without indicating segregation or forming additional phases.

## Figures and Tables

**Table 1:** Phase compositions were determined for the samples synthesized using different synthesis times.

Synthesis Time (h)	Phase Composition (%)	
	DCPA	Mg-WH
24	35	65
48	22	78
72	-	100

## Data Availability

Data are contained within the article.
